# Coexistence of Microscopic Polyangiitis, Systemic Lupus Erythematosus, and Transthyretin Amyloidosis in an Elderly Japanese Patient With Splenic Marginal Zone Lymphoma: An Autopsy Case Report

**DOI:** 10.7759/cureus.109085

**Published:** 2026-05-18

**Authors:** Yoshikuni Nagayama, Ayana Ichikura-Iida, Masako Otani, Tomonori Nakazato, Hiroyuki Hayashi

**Affiliations:** 1 Nephrology, Yokohama Municipal Citizen's Hospital, Yokohama, JPN; 2 Pathology, International University of Health and Welfare Mita Hospital, Tokyo, JPN; 3 Hematology, Yokohama Municipal Citizen’s Hospital, Yokohama, JPN; 4 Pathology, Yokohama Municipal Citizen's Hospital, Yokohama, JPN

**Keywords:** anca-associated vasculitis (aav), anti-neutrophil cytoplasmic antibody (anca), japanese geriatrics, marginal zone lymphoma (mzl), microscopic polyangiitis (mpa), sle (systemic lupus erythematosus), splenic marginal zone lymphoma (smzl), transthyretin amyloidosis (attr)

## Abstract

Marginal zone lymphoma (MZL) is a rare low-grade B-cell lymphoma characterized by its heterogeneous nature and association with chronic antigenic stimulation from infectious agents or autoimmune diseases. Both anti-neutrophil cytoplasmic antibody (ANCA)-associated vasculitis and systemic lupus erythematosus (SLE) are rarely associated with hematologic malignancies, including non-Hodgkin lymphoma. We herein report an 86-year-old man with splenic MZL (SMZL) in whom autopsy revealed the coexistence of microscopic polyangiitis (MPA), SLE, and transthyretin amyloidosis (ATTR). The patient was admitted for rapidly progressive renal dysfunction (serum creatinine (Cr) from 2.04 to 6.77 mg/dL over two months) with general fatigue and loss of appetite. One year previously, SMZL had been diagnosed based on bone marrow biopsy and clinical features. At that time, the patient had renal dysfunction (Cr, 1.77 mg/dL) due to nephrosclerosis. The treatment for SMZL was postponed due to the advanced age and stable condition. On admission, MPA was suspected based on rapidly progressive glomerulonephritis, mononeuritis multiplex, and myeloperoxidase (MPO)-ANCA positivity. Hemodialysis and glucocorticoids improved serum C-reactive protein and MPO-ANCA levels after three weeks, but anuria persisted, and the patient remained dialysis-dependent. Aspiration pneumonia temporarily improved with ceftriaxone sodium hydrate; however, the patient died of sudden deterioration of oxygenation on the 49th hospital day. An autopsy revealed crescentic nephritis complicated by lupus nephritis and ATTR in the lungs and heart. The direct cause of death was cardiac and respiratory failure due to ATTR. This case highlights the potential pathophysiological interplay of multiple immune-mediated and neoplastic conditions in elderly patients, underscoring the importance for clinicians to recognize that patients with SMZL may have coexisting autoimmune diseases such as MPA and SLE. In particular, in elderly patients, the possibility of occult ATTR should also be considered.

## Introduction

Marginal zone lymphoma (MZL) is an indolent B-cell lymphoma derived from marginal zone B-cells present in lymph nodes and extranodal tissues, with three distinct subtypes: splenic, nodal, and extranodal lymphoma, which collectively account for 5-17% of non-Hodgkin lymphomas [1]. Splenic MZL (SMZL) is diagnosed as a clinicopathologic entity from a combination of clinical features including splenomegaly, moderate lymphocytosis, and cytopenia, and peripheral smear findings, flowcytometric immunophenotyping, and morphological and immunohistochemical (IHC) findings in bone marrow biopsy [2]. The immunophenotype is usually that of a mature B-cell lymphoma: CD20/CD79a and CD79b are positive, while CD5, CD10, CD23, cyclin D1, annexin A1, CD11c, and CD25 are typically absent [2]. MZL is characterized by its heterogeneous nature and association with chronic antigenic stimulation from infectious agents or autoimmune diseases [3]. Microscopic polyangiitis (MPA) is usually diagnosed clinically as a pauci-immune necrotizing small-vessel vasculitis, most often with renal involvement. In the 2022 American College of Rheumatology/European Alliance of Associations for Rheumatology classification criteria for MPA [4], the final criteria and their weights were as follows: perinuclear antineutrophil cytoplasmic antibody (ANCA) or anti-myeloperoxidase-ANCA positivity (+6), pauci-immune glomerulonephritis (+3), lung fibrosis or interstitial lung disease (+3), sino-nasal symptoms or signs (−3), cytoplasmic ANCA or anti-proteinase 3 ANCA positivity (−1), and eosinophil count ≥1×10^9^/L (−4). After excluding mimics of vasculitis, a patient with a diagnosis of small- or medium-vessel vasculitis could be classified as having MPA with a cumulative score of ≥5 points. Patients with ANCA-associated vasculitis (AAV) have an increased risk of preceding or concurrent malignancy [5]. AAV is rarely associated with hematologic malignancies, mainly non-Hodgkin’s lymphoma and myelodysplasia [6]. The diagnosis of systemic lupus erythematosus (SLE) is based on the Systemic Lupus International Collaborating Clinics classification (SLICC) criteria [7]. According to the SLICC classification, the patient must satisfy at least four criteria, including at least one clinical criterion and one immunologic criterion, or the patient must have biopsy-proven lupus nephritis in the presence of antinuclear antibodies or anti-double-stranded DNA antibodies. Similarly, patients with SLE have an increased risk of cancer, especially hematologic malignancies, including non-Hodgkin lymphoma [8]. SLE patients with a preceding or concurrent hematologic malignancy often present with early-stage hematologic malignancies, regardless of the type of malignancy [9]. Amyloidosis is diagnosed pathologically by detecting amyloid deposition, followed by subtype classification of the deposited amyloid protein, through which transthyretin amyloidosis (ATTR) is diagnosed. ATTR is a progressive disease caused by the accumulation of mutated or normal transthyretin throughout the body, particularly affecting the heart and nervous system, which can be hereditary or age-related [10]. ATTR often presents as heart failure with preserved ejection fraction and has been historically underdiagnosed [10]. ATTR, particularly wild-type ATTR cardiomyopathy (ATTRwt-CM), is especially relevant in elderly Japanese patients because ATTRwt is fundamentally an age-related disease, and Japan has one of the world’s most rapidly aging populations [11]. The pathophysiological relationship between SMZL and ATTR remains unclear; however, both SMZL and ATTR are diseases strongly associated with aging, and therefore, aging may promote this coexistence. We herein report an elderly Japanese patient of SMZL in which autopsy revealed the coexistence of three distinct pathological conditions: MPA, SLE, and ATTR. Those three diagnoses were identified only at autopsy, underscoring the central clinical lesson that antemortem recognition of this constellation is exceptionally challenging and occult ATTR in particular may be missed.

## Case presentation

Medical history and initial laboratory data

**Figure 1:**
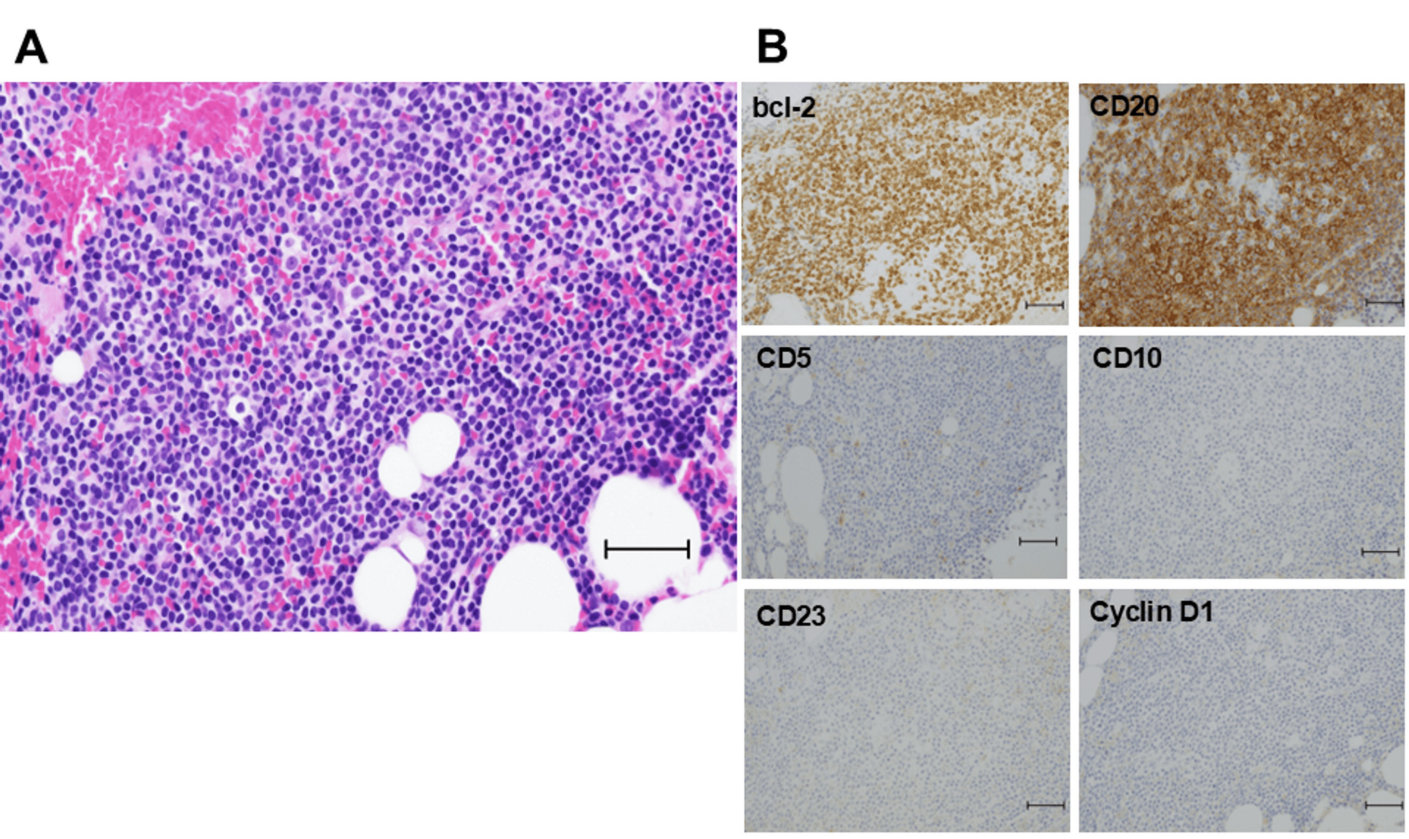
Examination of a needle biopsy of the bone marrow by light microscopy. (A) Hematoxylin and eosin staining section reveals that approximately 70% of the bone marrow cavity is occupied by nucleated cells, two-thirds of which consist of sheet-like aggregates of small lymphocytes. These lymphocytes have small, relatively uniform, and oval nuclei and have a translucent cytoplasm. (B) Immunohistochemical analysis reveals that the lymphocytes are positive for bcl-2 and CD20 and negative for CD5, CD10, CD23, and Cyclin D1, resulting in the diagnosis of marginal zone lymphoma. Bars = 200µm

An 86-year-old Japanese man was carried to the emergency department of our tertiary care hospital for general fatigue and loss of appetite for a few weeks. The patient was admitted to our department of nephrology for rapidly progressive renal dysfunction (serum creatinine (Cr), from 2.04 mg/dL to 6.77 mg/dL) in two months. One year previously, the patient was referred to the Department of Hematology for hyperleukocytosis. A bone marrow biopsy revealed that the patient had low-grade B-cell lymphoma (Figure 1A). Immunohistochemically, the lymphocytes were positive for bcl-2, CD20, and CD79a, and negative for CD3, CD5, CD10, CD23, and Cyclin D1, resulting in the diagnosis of MZL (Figure 1B).

Based on the clinical features of superficial lymphadenopathy, splenomegaly, and leukocytosis, the subtype of MZL was considered to be SMZL. At that time, the patient had a 16-year history of hypertension and renal dysfunction (Cr, 1.77 mg/dL) due to nephrosclerosis. The treatment for SMZL was postponed due to the advanced age and stable condition. The patient’s clinical findings on admission were as follows: consciousness, alert; blood pressure, 162/96 mmHg; pulse rate, 72/min; body temperature, 36.8 °C; percutaneous oxygen saturation at room, 96%. There was no superficial lymphadenopathy, joint pain, skin lesions, or lower extremity edema, and an examination of his heart and lungs was unremarkable. There was hepatosplenomegaly and multiple hypesthesias in his feet. Laboratory findings on admission are shown in Table 1.

**Table 1:** Laboratory findings on admission Ab, antibody; Ag, antigen; ALP, alkaline phosphatase; ALT, alanine aminotransferase; ANA, anti-nuclear antibody; ANCA, anti-neutrophil cytoplasmic antibody; AST, aspartate aminotransferase; β2-MG, beta-2 microglobulin; BUN, blood urea nitrogen; CL, cardiolipin; Cr, creatinine; CRP, C-reactive protein; ds-DNA, double strand-DNA ; eGFR, estimated glomerular filtration rate; GBM, glomerular basement membrane; γ-GTP, gamma-glutamyl transpeptidase; GP, glycoprotein; HbA1c, hemoglobin A1c; HBV, hepatitis B virus; HCV, hepatitis C virus; HIV, human immunodeficiency virus; HPF, high power field; IC, immune complex; IgA, immunoglobulin A; IgG, immunoglobulin G; IgM, immunoglobulin M; LDH, lactate dehydrogenase; LDL-Cho, low-density lipoprotein-cholesterol; MPO, myeloperoxidase; NAG, N-acetyl-β-D-glucosaminidase; PR3, proteinase 3; RF, rheumatoid factor; RNP, ribonucleoprotein; RPR, rapid plasma reagin; sIL-2R, soluble interleukin-2 receptor; ss-DNA, single strand-DNA; SS, Sjögren syndrome; STS; serologic test for syphilis; TG, triglyceride; WBC, white blood cell; WF, whole field.

Parameter	Value (reference range)
Hematology	
WBC count, /µL	14500 (3500-9000)
Band/Seg/Lymph, %	2/37/53
Mono/Eosino/Baso, %	5/0/1
Atyp. Lymph, %	2
Hemoglobin, g/dL	9.9 (12.9-17.4)
Platelet count, 10^4^/µL	8.3 (13-37)
Blood chemistry	
Cr, mg/dL	6.77 (0.52-1.15)
eGFR, mL/min/1.73m^2^	6.6
BUN, mg/dL	110 (8.0-21.0)
Total protein, g/dL	8.0 (6.7-8.3)
Albumin, g/dL	3.6 (3.8-5.3)
AST, U/L	18 (8-38)
ALT, U/L	11 (4-44)
LDH, U/L	182 (106-211)
ALP, U/L	195 (104-338)
γ-GTP, U/L	15 (0-60)
LDL-Cho, mg/dL	103 (<140)
TG, mg/dL	84 (55-150)
Glucose, mg/dL	174 (70-110)
HbA1c, %	5.4 (4.9-6.0)
CRP, mg/dL	1.6 (<0.14)
Urinalysis	
Urine dipstick protein	3+
Microscopic hematuria	3+
Red blood cells	>100/HPF
Granular casts	1-4/WF
Urinary protein, g/day	1.3 (<0.15)
β2-MG, µg/L	1132 (<250)
NAG, IU/L	24.4 (<10)
M protein	Negative
Immunology	
IgG, mg/dL	1780 (640-1800)
IgA, mg/dL	188 (70-350)
IgM, mg/dL	90 (40-290)
M protein	IgM-λ positive
C3, mg/dL	71 (80-140)
C4, mg/dL	39.7 (11-34)
CH_50_, U/mL	29 (30-45)
C1q-IC, µg/mL	6.8 (0.0-2.9)
RF, U/mL	202 (<15)
Cryoglobulin	Negative
Serum amyloid A, µg/mL	56.5 (0.0-8.0)
ANA, titer	320 (<80)
Anti-ds-DNA Ab, IU/mL	23.8 (0.0-19.9)
Anti-ss-DNA Ab, AU/mL	>800 (0.1-39.9)
Anti-CL IgG, U/mL	2 (0-9)
Anti-CLβ2 GP1, U/mL	<1.3 (0.0-3.4)
Anti-SS-A/B Ab	Negative/negative
Anti-Smith Ab	Negative
Anti-RNP Ab	Negative
MPO-ANCA, EU	69 (0-19)
PR3-ANCA, EU	<10 (0-9)
Anti-GBM Ab	Negative
sIL-2R, U/mL	4738 (204-587)
Ferritin, ng/mL	142.7 (21.0-282)
HBV surface Ag	Negative
HCV Ab	Negative
STS (RPR method)	Positive
Treponema pallidum Ab	Negative
HIV Ab	Negative

A urinalysis revealed protein 3+, blood 3+, sediment of white blood cells (10 to 19 per high-power field), red blood cells (over 100 per high-power field), hyaline casts (1 to 4 per whole field), and granular casts (1 to 4 per whole field), elevated urinary protein (1.3 g/day), elevated β2-microglobulin, and elevated N-acetyl-β-D-glucosaminidase. Blood tests revealed hyperleukocytosis, anemia, thrombocytopenia, hypoalbuminemia, renal dysfunction, and elevated C- reactive protein (CRP). The eGFR was calculated using the Japanese eGFR equation (eGFR = 194× Cr ^− 1.094^ × age ^− 0.287^) [12]. Serum IgG, IgM, and IgA were within the normal range, although a small amount of IgM-λtype M protein was detected by immune-electrophoresis. Both serum soluble interleukin-2 receptor and amyloid A protein were elevated. Serum C3 and CH50 were slightly low with normal serum C4. Myeloperoxidase anti‐neutrophil cytoplasmic antibody (MPO-ANCA) was positive. The anti-nuclear antibody (ANA) test was positive. Anti-double-strand DNA antibody and anti-single-strand DNA antibody were positive. Anti-cardiolipin (CL) IgG and anti-CLβ2 glycoprotein 1 complex were negative. Rheumatoid factor and C1q-immune complex were positive. The patient lacked proteinase 3-ANCA, anti‐glomerular basement membrane antibody, anti-SS-A and B antibodies, anti-Smith antibody, anti-RNP antibody, and cryoglobulin. The patient had no infectious diseases, including hepatitis B virus, hepatitis C virus, and human immunodeficiency virus. The serological test for syphilis was a biological false positive. The ANA positivity, positive anti-dsDNA and anti-ssDNA antibodies, low C3/CH50 levels, elevated C1q immune complexes, and biologically false-positive serologic test for syphilis collectively constituted a serological signature of SLE that was not initially recognized as such. There were no abnormalities on chest radiography or an electrocardiogram. Echocardiographic M-mode findings showed a left ventricular end-diastolic dimension of 44 mm, left ventricular end-systolic dimension of 28.1 mm, interventricular septal thickness of 9.4 mm, posterior wall thickness of 10.5 mm, and a left ventricular ejection fraction of 66%, suggesting mild concentric remodeling with preserved systolic function. The peripheral nerve conduction studies revealed prolonged distal latency in the left median nerve, suggesting the presence of carpal tunnel syndrome. In addition, compound muscle action potential (CMAP) was not elicitable in the ulnar and peroneal nerves, while the elicited CMAP in the tibial nerve showed reduced amplitudes and temporal dispersion, consistent with mononeuritis multiplex with mixed axonal and demyelinating features. Abdominal computed tomography revealed swelling of both kidneys and splenomegaly.

Clinical course

**Figure 2:**
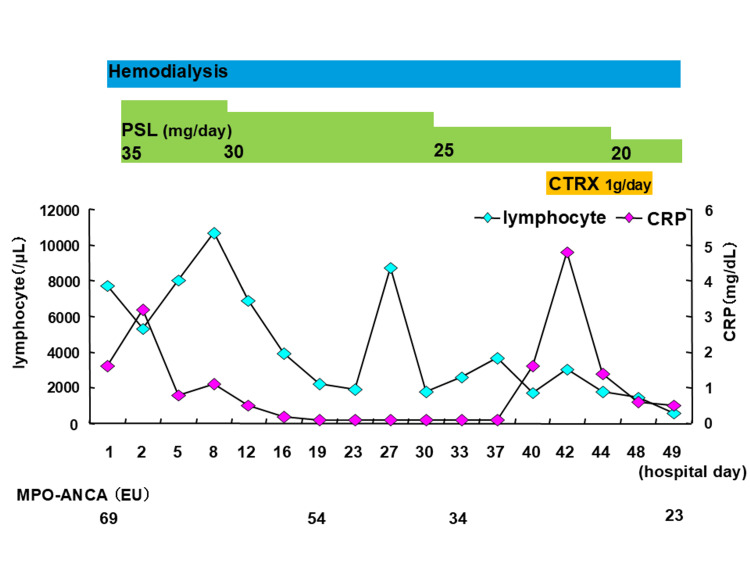
The clinical course of the patient Microscopic polyangiitis was suspected, and hemodialysis and prednisolone (PSL) were initiated. The serum C-reactive protein (CRP) level was negative, and myeloperoxidase anti-neutrophil cytoplasmic antibody (MPO-ANCA) titer was decreased; however, anuria persisted, and the patient became dialysis-dependent. On the 40th hospital day, the serum CRP level became elevated, and aspiration pneumonia was suspected based on chest radiography. Ceftriaxone sodium hydrate (CTRX) was initiated, leading to a subsequent improvement in pneumonia and a decrease in the serum CRP level, allowing for discontinuation of CTRX. However, the patient died of sudden deterioration of oxygenation on the 49th day of hospitalization.

Considering the combination of rapidly progressive glomerulonephritis, mononeuritis multiplex, and MPO-ANCA positivity, MPA was suspected [13]. Hemodialysis and glucocorticoids (35 mg/day of intravenous prednisolone) were initiated. Three weeks later, the serum CRP level was negative, and MPO-ANCA titer was decreased (Figure 2).

However, anuria persisted, and the patient became dialysis-dependent. On the 40th hospital day, the serum CRP level became elevated, and aspiration pneumonia was suspected based on chest radiography. Ceftriaxone sodium hydrate (CTRX) was initiated, leading to subsequent improvement in pneumonia and a decrease in the serum CRP level, allowing for discontinuation of CTRX (Figure 2). However, the patient died of sudden deterioration of oxygenation on the 49th day of hospitalization.

Pathological findings

**Figure 3:**
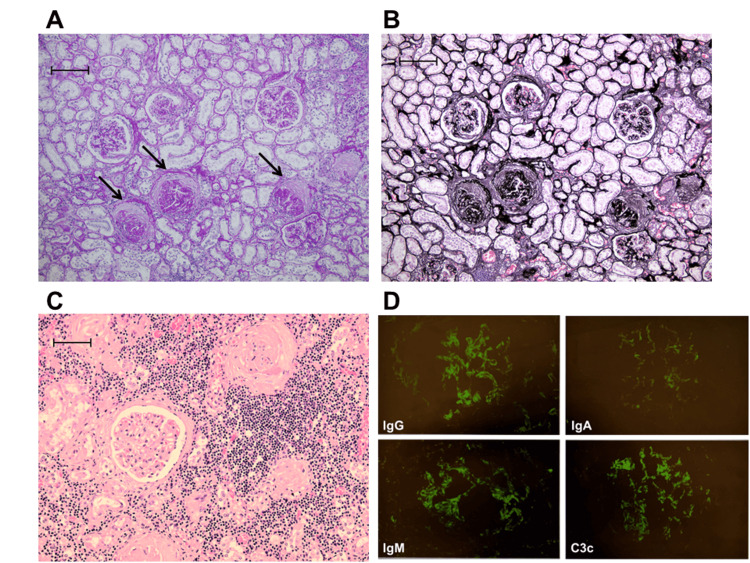
Examination of a kidney autopsy specimen by light microscopy and immunofluorescence microscopy Periodic-acid-Schiff (PAS) staining section (A) and periodic-acid-silver-methenamine staining section (B) show four glomeruli with global mesangial proliferative glomerulonephritis and three glomeruli with fibrocellular crescentic glomerulonephritis (arrows). A and B show the same cortical area. Hematoxylin and eosin staining section (C) shows atypical lymphocyte infiltration in the renal cortical interstitium. Immunofluorescence (D) reveals strong IgG, IgM, and C3c and weak IgA expression in the mesangium. Bars = 200 µm for A and B, and 100 µm for C.

An autopsy was performed after obtaining consent from the patient’s family. Pathological findings revealed that atypical lymphocytes infiltrated several organs, including bone marrow, spleen, liver, and kidneys. The glomeruli showed diffuse global fibrocellular crescents with mesangial matrix increase (Figures 3A, 3B).

**Figure 4:**
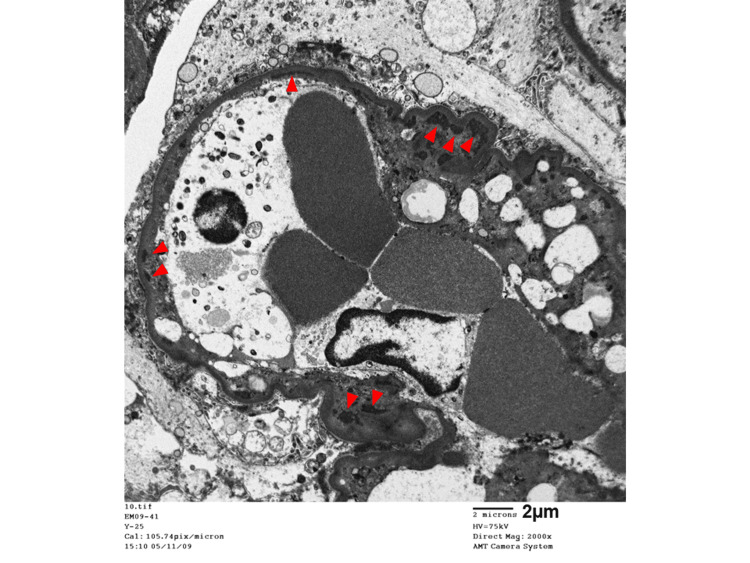
Examination of a kidney autopsy specimen by electron microscopy There are subendothelial, mesangial, and paramesangial electron-dense deposits (arrowheads).

Among 179 glomeruli observed, 79 showed crescentic formation, including sclerotic glomeruli. Mesangial cell proliferation in the non-sclerotic glomeruli was mild. The renal cortical interstitium showed atypical lymphocyte infiltration (Figure 3C). Immunofluorescence revealed strong IgG, IgM, and C3c, and weak IgA expression in the mesangium (Figure 3D). C1q staining was not performed. Electron microscopy revealed subendothelial, mesangial, and paramesangial electron-dense deposits (Figure 4).

**Figure 5:**
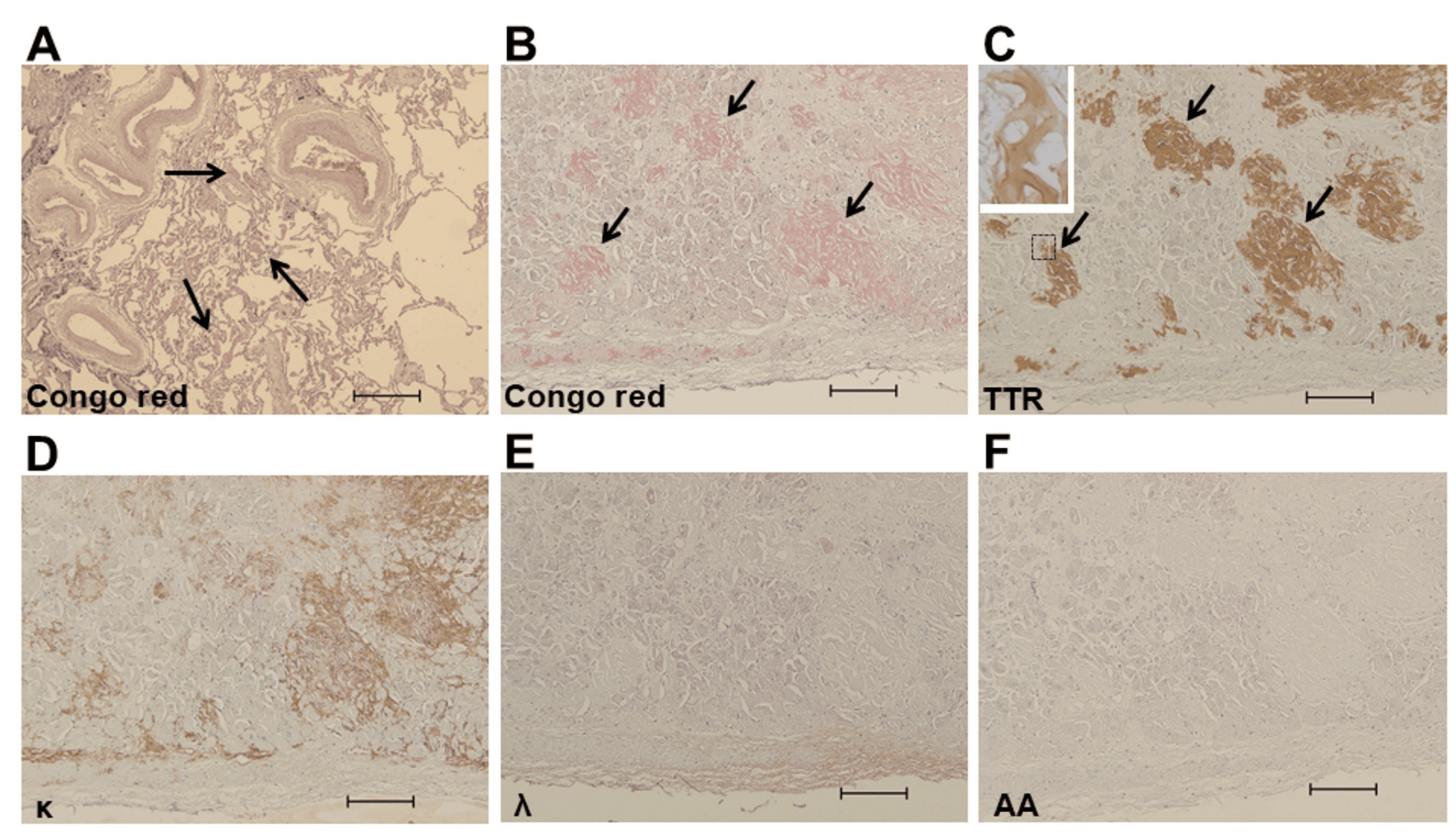
Examination of a lung and a heart autopsy specimen by light microscopy Congo red staining reveals amyloid deposits in the alveolar septum of the lung (A) (arrows) and in the endocardium and cardiomyocytes (B) (arrows). The immunohistochemical analysis reveals that the deposition of transthyretin (TTR) is positive (C) (arrows). A dot square is enlarged on a higher- magnification inset (C). κ light chain (D) shows non-specific positive staining. λ light chain (E) and amyloid A (F) are negative. B to F show the same area in the heart. Bars = 250 µm for A, and 100 µm for B to F.

Congo red staining revealed amyloid deposits in several organs, including the lungs (Figure 5A), heart (Figure 5B), prostate, urinary bladder, and adrenal glands.

In the lungs, amyloid deposition was significant in the alveolar septum. In the heart, widespread amyloid deposits were found in the endocardium and cardiomyocytes. Furthermore, amyloid precursor proteins were determined by IHC analysis. In the heart, IHC analysis reveals that the deposition of transthyretin is positive (Figure 5C). κ light chain (Figure 5D) shows non-specific positive staining. Because the sites of transthyretin positivity showed the closest concordance with the areas of amyloid deposition, the κ light chain staining was considered to represent non-specific positive staining. λ light chain (Figure 5E) and amyloid A (Figure 5F) are negative. In the kidneys, amyloid deposits were not found. The direct cause of death was respiratory failure due to cardiac and diffuse alveolar septal amyloidosis.

## Discussion

The present patient was suspected of having MPA during hospitalization, and the postmortem pathological examination confirmed MPA and additionally revealed the coexistence of SLE and ATTR. SLE was not listed in our differential diagnosis before the autopsy; however, we reviewed the clinical and pathological data, based on the SLICC criteria [7], leading to the diagnosis of SLE. The patient met at least four items, including two clinical items (proteinuria and thrombocytopenia) and two immunologic items (higher titer of serum ANA and low level of serum complement). Moreover, the autopsy-proven lupus nephritis was confirmed. The patient also fulfilled the 2019 EULAR/ACR classification criteria for SLE, with a total score of ≥10 based on proteinuria, renal biopsy findings, low C3 levels, and positivity for anti-dsDNA antibody [14]. In fact, at the diagnosis of SMZL one year previously, the patient already had some clinical characteristics suggesting SLE as follows: anemia, low serum complement levels (C3, 73 mg/dL, reference range: 80-140 and CH50, 24 U/mL, reference range: 30-45), anti-DNA antibody-positive, (41.9 IU/mL, reference range: 0.0-6.0), and C1q-immune complex-positive (7.2 µg/mL, reference range: 0.0-2.9); However, urinalysis had not been performed, making it unclear when proteinuria appeared. Therefore, it remained uncertain whether the patient met the SLICC criteria for SLE.

In the present patient, SMZL was complicated by autoimmune diseases such as MPA and SLE. The potential role of malignancy as a trigger for AAV remains inconclusive, with limited evidence supporting this hypothesis [15]. Some retrospective observational studies supported the hypothesis [6], while others did not [16,17]. The association between AAV and malignancy may be multifactorial, involving impaired immunosurveillance, oncogenic effects of immunosuppressive treatments, and chronic immune stimulation [15]. Mixed cryoglobulinemic vasculitis is frequently reported as vasculitis associated with MZL [18]. However, there are a few cases of MZL complicated by MPA, as seen in the present case. On the other hand, a large international cohort study found elevated standardized incidence ratios for overall cancer, hematologic malignancies, and non-Hodgkin lymphoma in SLE patients [8]. Potential mechanisms underlying this association between SLE and malignancy include chronic immune dysregulation, immunosuppressant drugs, genetic factors, and environmental exposures [19]. However, the exact nature of the SLE-cancer link remains controversial. Although the association between SMZL and the coexistence of MPA and SLE in this case remains unclear, careful follow-up considering the potential coexistence of autoimmune diseases such as MPA and SLE in patients with SMZL may be warranted.

Treatment strategies of SMZL are often individualized due to the rarity of SMZL and the lack of standardized guidelines, with options ranging from watchful waiting to chemotherapy and targeted therapies [1]. Recent advancements include the use of Bruton's tyrosine kinase inhibitors and other novel agents, which show promise in relapsed cases [1].

Based on the renal pathological autopsy findings, the rapid decline in renal function of the present patient was attributed to multiple factors, including MPA, lupus nephritis, and SMZL infiltration; however, approximately 44% (79/179) of the observed glomeruli showed crescentic formation, including sclerotic glomeruli. Mesangial cell proliferation in the non-sclerotic glomeruli was mild, and MPA was considered the primary contributor to the rapid renal dysfunction. Given the advanced age and the high risk of infection, glucocorticoid monotherapy was chosen as the treatment for MPA; however, the patient became dialysis-dependent. The addition of rituximab and/or plasma exchange might have been beneficial for the renal prognosis of the present patient [20].

The autopsy revealed that ATTR predominantly affected the lungs and heart. Genetic testing was unavailable, and we were not able to evaluate any pathological features favoring wild-type ATTR over hereditary ATTR; therefore, it remained unclear whether ATTR in the present case was hereditary or wild-type. In Japan, *Val30Met* is the most common pathogenic variant associated with hereditary ATTR, and endemic foci have been reported in Nagano and Kumamoto prefectures; however, late-onset cases arising in non-endemic areas have also been described [21]. The patient was born in Nagano prefecture, and although there was no apparent family history, hereditary ATTR could not be excluded. In the most recent epidemiological study of systemic amyloidosis in Japan, ATTR was identified as the most prevalent subtype (54.9%) [22]. Analysis of the autopsy specimens also yielded similar findings, with ATTR being the predominant pathology; wild-type ATTR accounted for the largest proportion at 29.1% [23]. In contrast, the prevalence of ATTR was 4.1% in the 1990s, indicating a marked increase in its frequency in the Japanese population [24]. Population aging in Japan may have contributed to the increased prevalence of wild-type ATTR. In the present case, the terminal event was likely multifactorial in the context of multiple advanced organ-level pathologies including ATTR cardiomyopathy, pulmonary amyloidosis, residual pneumonia, uremic complications, and glucocorticoid-related immunosuppression; however, the autopsy revealed that the direct cause of death was cardiac and respiratory failure due to ATTR. Diagnosing ATTR during the patient’s lifetime was challenging. Troponin, NT-proBNP, or technetium pyrophosphate scintigraphy as a standard non-invasive tool for ATTR workup was unfortunately performed. In retrospect, the findings suggestive of ATTR during the patient’s lifetime included mild left ventricular hypertrophy on echocardiography and carpal tunnel syndrome detected by nerve conduction studies; however, both were clinically subtle, reinforcing the occult nature of ATTR. Given the aging population associated with increased longevity, ATTR should be considered to be a potential common disease.

## Conclusions

The present case was diagnosed at autopsy as SMZL complicated by MPA, SLE, and ATTR. The direct cause of death was cardiac and respiratory failure due to ATTR. Because this is a single case, the present observations remain hypothesis-generating rather than definitive; however, this case will provoke clinicians to recognize that patients with SMZL can have the coexistence of autoimmune diseases such as MPA and SLE. In particular, in elderly patients with SMZL, especially in the Japanese population where wild-type ATTR is increasingly prevalent, the possibility of occult ATTR should also be considered, and screening with echocardiography, cardiac biomarkers, and scintigraphy may be warranted.
